# Application of Artificial Neural Networks to Investigate One-Carbon Metabolism in Alzheimer’s Disease and Healthy Matched Individuals

**DOI:** 10.1371/journal.pone.0074012

**Published:** 2013-08-12

**Authors:** Fabio Coppedè, Enzo Grossi, Massimo Buscema, Lucia Migliore

**Affiliations:** 1 Department of Translational Research and New Technologies in Medicine and Surgery, Division of Medical Genetics, University of Pisa, Pisa, Italy; 2 Bracco Foundation, Milan, Italy; 3 Semeion Research Center, Rome, Italy; 4 Department of Mathematical and Statistical Sciences, University of Colorado at Denver, Denver, Colorado, United States of America; University of Pecs Medical School, Hungary

## Abstract

Folate metabolism, also known as one-carbon metabolism, is required for several cellular processes including DNA synthesis, repair and methylation. Impairments of this pathway have been often linked to Alzheimer’s disease (AD). In addition, increasing evidence from large scale case-control studies, genome-wide association studies, and meta-analyses of the literature suggest that polymorphisms of genes involved in one-carbon metabolism influence the levels of folate, homocysteine and vitamin B12, and might be among AD risk factors. We analyzed a dataset of 30 genetic and biochemical variables (folate, homocysteine, vitamin B12, and 27 genotypes generated by nine common biallelic polymorphisms of genes involved in folate metabolism) obtained from 40 late-onset AD patients and 40 matched controls to assess the predictive capacity of Artificial Neural Networks (ANNs) in distinguish consistently these two different conditions and to identify the variables expressing the maximal amount of relevant information to the condition of being affected by dementia of Alzheimer’s type. Moreover, we constructed a semantic connectivity map to offer some insight regarding the complex biological connections among the studied variables and the two conditions (being AD or control). TWIST system, an evolutionary algorithm able to remove redundant and noisy information from complex data sets, selected 16 variables that allowed specialized ANNs to discriminate between AD and control subjects with over 90% accuracy. The semantic connectivity map provided important information on the complex biological connections among one-carbon metabolic variables highlighting those most closely linked to the AD condition.

## Introduction

**Figure 1:**
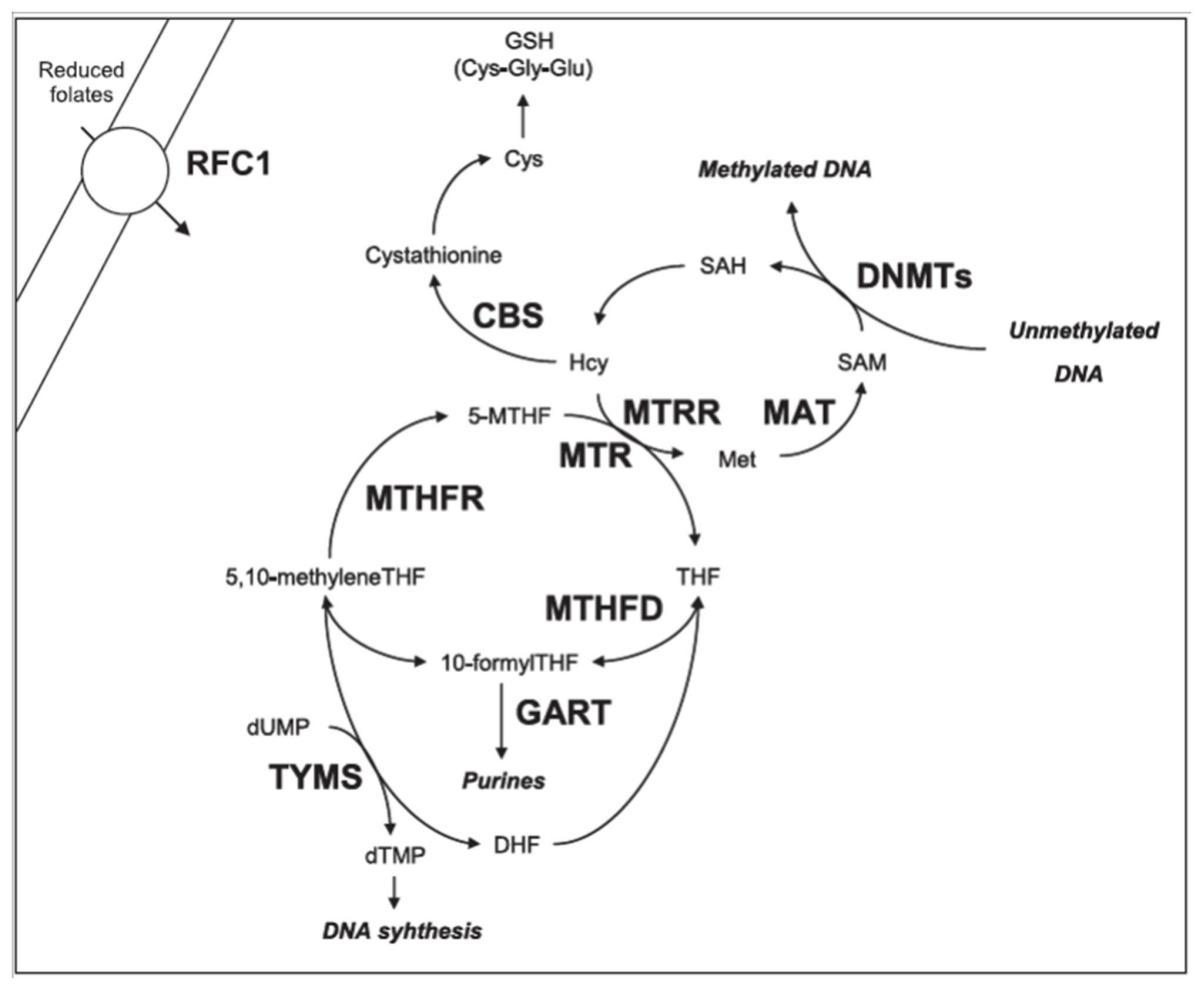
Overview of the folate metabolic pathway, adapted from [18]. Folates require several transport systems to enter the cells, the best characterized being the reduced folate carrier (RFC1). Methylenetetrahydrofolate reductase (MTHFR) reduces 5,10-methylenetetrahydrofolate (5,10-MTHF) to 5-methyltetrahydrofolate (5-MTHF). Subsequently, methionine synthase (MTR) transfers a methyl group from 5-MTHF to homocysteine (Hcy) forming methionine (Met) and tetrahydrofolate (THF). Methionine is then converted to S-adenosylmethionine (SAM) in a reaction catalyzed by methionine adenosyltransferase (MAT). Most of the SAM generated is used in transmethylation reactions, whereby SAM is converted to S-adenosylhomocysteine (SAH) by DNA methyltransferases (DNMTs) that transfer the methyl group to the DNA. Vitamin B12 is a cofactor of MTR, and methionine synthase reductase (MTRR) is required for the maintenance of MTR in its active state. If not converted into methionine, Hcy can be used for the synthesis of glutathione (GSH) in a reaction catalyzed by cystathionine b-synthase (CBS) and other enzymes. Another important function of folate derivatives (THF and dihydrofolate: DHF) is in the de novo synthesis of DNA and RNA precursors (dUMP, dTMP, etc). This pathway is mediated by thymidylate synthase (TYMS), methylenetetrahydrofolate dehydrogenase (MTHFD), and phosphoribosylglycinamide transformylase (GART) enzymes.

Folate metabolism, also known as one-carbon metabolism, plays a fundamental role in DNA synthesis and integrity, in chromosome stability, in DNA and protein methylation, as well as in antioxidant defence mechanisms, and impairments of this pathway have been often linked to Alzheimer’s disease (AD) risk [1–4]. In 1990, Regland and colleagues first reported elevated homocysteine (hcy) levels in patients with primary degenerative dementia [5]. Since then, several researchers have investigated the levels of hcy, folate, and other B group vitamins involved in one-carbon reactions, such as vitamin B12, in mild cognitive impairment and AD [6–8]. Most of the retrospective studies focusing on the comparison between plasma hcy levels in AD patients and healthy controls revealed increased hcy values in AD subjects [3,9]. Also evidence from prospective studies suggests that a moderate elevation in hcy levels is a potential AD risk factor [3,9]. However, results are often conflicting, and it remains controversial whether hyperhomocysteinemia (hhcy) is really an AD risk factor or rather a consequence of the disease [4,9,10]. Several retrospective studies observed significantly decreased serum folate levels in AD subjects with respect to controls, and an inverse correlation between plasma hcy and serum folate [1,11]. There is also indication that low serum vitamin B12 levels are associated with neurodegenerative diseases and cognitive impairment [12], and several clinical investigations have demonstrated that folate and related B-vitamins administration is able to reduce hcy levels and antagonize some mechanisms favouring neurodegenerative impairments, as mild cognitive impairment and dementia [13]. In addition, increasing evidence from large scale case-control studies, genome-wide association studies (GWAS), and meta-analyses of the literature suggest that polymorphisms of genes involved in one-carbon metabolism influence the levels of folate, hcy and vitamin B12, and might be among AD risk factors [8,14–17]. Unfortunately, the overall results of the literature are sometimes conflicting and often insufficient to disclose the effective relationship among studied variables [2]. This is partially due to the complexity of the one-carbon metabolic pathway (Figure 1) and to the number of genes and environmental factors involved [2], as well as to the fact that traditional statistical algorithms are both unsuitable and underpowered to dissect the relationship between high number of markers due to the non-linearity and complexity of the folate metabolic pathway [18].

We performed the present study using Artificial Neural Networks (ANNs) to identify key factors linking folate metabolism to AD. The method used by ANNs aims to understand natural processes and recreate those processes using automated models. These networks allow a method of forecasting with understanding of the relationship among variables, and in particular nonlinear relationships [19–21]. ANNs function by initially learning a known set of data from a given problem with a known solution (training) and then the networks, inspired by the analytical processes of the human brain, are able to reconstruct the imprecise rules which may be underlying a complex set of data (testing). In recent years ANNs have been used successfully in medicine, for example they have been used to investigate the predictive values of risk factors on the conversion of amnestic mild cognitive impairment to AD [22], to identify genetic variants essential to differentiate sporadic amyotrophic lateral sclerosis cases from controls [23,24], to understand the relationship among polymorphisms of genes involved in one-carbon metabolism, chromosome damage, and maternal risk for having a birth with Down syndrome [18], to detect multiple genes of smaller effects in predisposing individuals to Barrett’s esophagus [25], and to differentiate fronto-temporal dementia from AD [26], among others.

In this pilot study we applied ANNs to investigate biochemical and genetic markers related to one-carbon metabolism in 40 late onset AD patients and 40 matched controls selected from a previously described database [8,27] in order to assess the predictive capacity of ANNs in distinguish consistently these two different conditions and to identify the variables expressing the maximal amount of relevant information. Moreover, we used the Auto Contractive Map-Auto-CM algorithm (Auto-CM), a special kind of Artificial Neural Network able to define the strength of the associations of each variable with all the others and to visually show the map of the main connections of the variables and the basic semantic of their ensemble [28,29]. Auto-CM was previously applied by us to a dataset of genetic and cytogenetic data collected from mothers of Down syndrome individuals and matched control mothers [18] and successfully disclosed previously unknown connections among polymorphisms of genes involved in folate metabolism and chromosome damage and malsegregation events in those women.

At best of our knowledge no previous study has investigated the relationship among biochemical markers of one-carbon metabolism (folate, hcy, vitamin B12) and genetic polymorphisms of major enzymes involved in this pathway (methylenetetrahydrofolate reductase: MTHFR; methionine synthase: MTR; methionine synthase reductase: MTRR; thymidylate synthase: TYMS; reduced folate carrier: RFC1; DNA methyltransferases: DNMTs) by means of ANNs in AD. The aim of this study was to investigate whether this revolutionary mathematical approach can increase our knowledge on the connections among those variables in AD and matched control individuals and to identify key variables to discriminate among these two conditions.

## Materials and Methods

### Study Population

**Figure 2:**
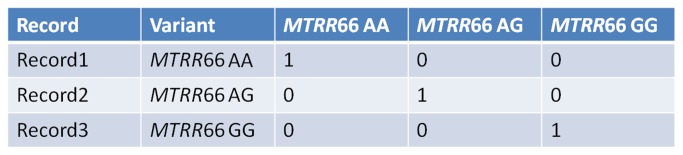
Method of coding the polymorphisms in the database. The code assigned to the polymorphisms transformed each polymorphism in three genotype classes: wild type (major homozygous), heterozygous and mutants (minor homozygous). For each class a binary coding was applied: 0 if variable absent; 1 if variable present. So for example considering the polymorphism *MTRR* 66A>G which can exist in three variants: AA (major homozygous), AG (heterozygous) and GG (minor homozygous). Supposing that three records are AA, GG and AG, the coding has been applied as shown in the figure.

We aimed to re-analyze from a completely new perspective some of the data obtained from our previous studies [8,27]. From a previously described dataset [8,27] containing data from AD patients and healthy matched controls, we have selected 40 late onset AD (15 males and 25 females, mean age at sampling 78.1 ± 6.3 years) and 40 age and sex matched control subjects (17 males and 23 females, mean age at sampling 76.5 ± 6.7 years) for whom all the following information on one-carbon metabolism was available: 1) plasma hcy levels, 2) serum folate levels, 3) serum vitamin B12 levels, 4) genotype for the *MTHFR* 677C>T (CC, CT or TT) polymorphism (rs1801133), 5) genotype for the *MTHFR* 1298A>C (AA, AC or CC) polymorphism (rs1801131), 6) genotype for the *MTRR* (AA, AG or GG) 66A>G polymorphism (rs1801394), 7) genotype for the *MTR* 2756A>G (AA, AG or GG) polymorphism (rs1805087), 8) genotype for the *SLC19A1* (*RFC1*) 80G>A (AA, AG, GG) polymorphism (rs1051266), 9) genotype for *TYMS* 28-bp repeats (2R2R,2R3R,3R3R) polymorphism (rs34743033), 10) genotype for *TYMS* 1494 6-bp ins/del (+/+, +/-, -/-) polymorphism (rs34489327), 11) genotype for *DNMT3B* -149C>T (CC, CT, TT) polymorphism (rs2424913), and 12) genotype for *DNMT3B* -579G>T (GG, GT, TT) polymorphism (rs1569686). As detailed elsewhere [8,27] all subjects included in our dataset were Caucasians of Italian origin (North-West Tuscany and neighboring areas) and diagnosis of probable AD was performed according to DSM-IV and NINCDS-ADRDA criteria at the time of patients recruitment [8,27]. The 40 AD subjects included in the present study also met the revised core criteria for probable AD [30]. A progressive cognitive decline on subsequent evaluations was observed. All the subjects included in the present study were sporadic cases, and none of them was a carrier of a causative genetic mutation in *APP*, *PSEN1*, or *PSEN2* [30]. Control subjects consist of healthy volunteer subjects having no individual or family history of dementia or cognitive decline [8,27]. Table 1 shows the distribution of the studied variables among AD subjects and controls. All the samples were coded and data were processed in blind by operators. Figure 2 explains how genotypes were coded in the database. All individuals gave written informed consent for inclusion in the database, whose creation was performed in accordance with the Helsinki Declaration and approved by the Ethics Committee of the Pisa University Hospital (Project Reference N° 3618/2012).

**Table 1:** Distribution of selected variables among cases and controls.

	**Alzheimer**		**Controls**		***P*-value**
**Parameter**	Mean	95% C.I.	Mean	95% C.I.	
Folates (ng/ml)	6.2	1.8	6.8	1.2	N.S.
Homocysteine (μmol/l)	22.3	4.7	16.2	1.7	<0.01
Vitamin B12 (pg/ml)	401.3	78.2	404.9	73.5	N.S
*MTHFR*_C677T_wild_type (CC)	28%	14%	38%	16%	N.S
*MTHFR*_C677T_heterozygous (CT)	40%	16%	47%	16%	N.S
*MTHFR*_C677T_mutant (TT)	32%	15%	15%	12%	N.S
*MTHFR*_A1298C_wild_type (AA)	45%	16%	60%	16%	N.S
*MTHFR*_A1298C_heterozygous (AC)	55%	16%	40%	16%	N.S
*MTHFR*_A1298C_mutant (CC)	0%	0%	0%	0%	N.S
*TYMS*_28bp_wild_type (2R2R)	32%	15%	20%	13%	N.S
*TYMS*_28bp_heterozygous (2R3R)	45%	16%	57%	16%	N.S
*TYMS*_28bp_mutant (3R3R)	23%	14%	23%	14%	N.S
*TYMS*_6bp_wild_type (+/+)	15%	12%	35%	15%	N.S
*TYMS*_6bp_heterozygous (+/-)	57%	16%	53%	16%	N.S
*TYMS*_6bp_mutant (-/-)	28%	14%	13%	11%	N.S
*MTRR*_A66G_wild_type (AA)	23%	14%	25%	14%	N.S
*MTRR*_A66G_heterozygous (AG)	43%	16%	52%	16%	N.S
*MTRR*_A66G_mutant (GG)	35%	15%	23%	14%	N.S
*MTR*_A2756G_wild_type (AA)	82%	12%	87%	11%	N.S
*MTR*_A2756G_heterozygous (AG)	15%	12%	13%	11%	N.S
*MTR*_A2756G_mutant (GG)	3%	5%	0%	0%	N.S
*RFC1*_A80G_wild_type (AA)	22%	14%	10%	10%	N.S
*RFC1*_A80G_heterozygous (AG)	60%	16%	62%	16%	N.S
*RFC1*_A80G_mutant (GG)	18%	12%	28%	14%	N.S
*DNMT3B*-149C>T_wild_type (CC)	45%	16%	50%	16%	N.S
*DNMT3B*-149C>T_heterozygous (CT)	47%	16%	40%	16%	N.S
*DNMT3B*-149C>T_mutant (TT)	8%	9%	10%	10%	N.S
*DNMT3B*-5799G>T_wild_type (GG)	48%	16%	62%	16%	N.S
*DNMT3B*-579G>T_heterozygous (GT)	47%	16%	28%	14%	N.S
*DNMT3B*-5799C>T_mutant (TT)	5%	7%	10%	10%	N.S

### Genotyping and biochemical data collection.

The database data concerning folate, hcy and vitamin B12 values and the genotypes for all the studied polymorphisms have been previously obtained by means of standard diagnostic protocols and validated PCR/RFLP techniques as described elsewhere [8,27].

### Mathematical methods

To evaluate a possible correlation between the studied variables (Table 1) and AD we have trained different learning machines [31–45] (listed in Table 2) using two validation protocols: the Training and Testing with random split and the K-Fold Cross Validation (K=10). Most of the used learning machines [31–43] are available on the WEKA data mining software [46], developed at the University of Waikato, New Zealand, while two ANNs (Self Momentum Back Propagation and Sine Net) [44,45] were implemented in “Supervised ANNs Software”, developed at the Semeion Research Center in Rome, Italy (Buscema M (1999-2010) Supervised ANNs. Semeion software #12, version 16.0). However, there is a lot of scientific literature about the pruning algorithms as necessary pre-processing tools able to eliminate noisy variables before the main test of pattern recognition [47–50]. Noisy input attributes sometime can hide the small meaningful information embedded in other attributes. To verify this possibility we used a special and a powerful input selection algorithm, named TWIST (Training With Input Selection and Testing), recently published [51], and developed in a special research software at the Semeion Research Center in Rome, Italy (Buscema M (2006-2012) TWIST Input Search, Semeion software #39, version 3.2).

**Table 2:** Learning Machine used in this application.

**Learning Machine**	**Nick Name**	**References**	**Software Package**
AdaBoostM1	AdaBoost	[30]	WEKA
Bagging	Bagging	[31]	WEKA
BayesNet	BayesNet	[33]	WEKA
KNN	IBk	[35]	WEKA
C4.5	J48	[36]	WEKA
KStar	KStar	[37]	WEKA
Logistic	Logistic	[38]	WEKA
LogitBoost	LogitBoost	[39]	WEKA
MultiLayer Perceptron	MLP	[40]	WEKA
NaivBayes	NaivBayes	[34]	WEKA
RandomForest	RandomForest	[32]	WEKA
RotationForest	RotationForest	[41]	WEKA
Sequential Minimal Optimization	SMO	[42]	WEKA
Self Momentum BackPropagation	FF_BP	[43]	Semeion
Sine Net	FF_SN	[44]	Semeion

### TWIST algorithm

TWIST algorithm is a complex evolutionary algorithm able to look for the best distribution of the global dataset divided in two optimally balanced subsets containing a minimum number of input features useful for optimal pattern recognition. TWIST is an evolutionary algorithm based on a seminal paper about Genetic Doping Systems [52], already applied to medical data with very promising results [18,19,21,53–59]. Usually TWIST evolutionary system is constituted by a population of Multilayer Perceptrons. Each ANN has to learn a subset of the global dataset and has to be tested in a blind way with another subset. In this application we re-program the fitness function of TWIST: we exchange the population of Multilayer Perceptrons with a population of simple K Nearest Neighbour (KNN), based on Euclidean metric. This change makes TWIST faster and more oriented to discover explicit similarities between input attributes and classes (AD and Controls). And that is exactly what we were looking for. Indeed, TWIST selected 16 of the 30 original attributes (see Table 3) and generated a global dataset of 16 attributes, and two optimal subsets for training and testing. We then applied the K-Fold protocol to the global dataset to verify if the 16 attributes selected by TWIST may improve the performances of the learning machines already applied to the original dataset. Moreover, since the K-fold protocol is not always a trustable strategy [51], as a second step we have applied the same learning machines (Table 2) to the two subsets generated directly by TWIST.

**Table 3:** The 16 variables selected by TWIST algorithm.

**Original Input = 30**	**Input Seletced by TWIST = 16**
Folates	Folates
Homocysteine	Homocysteine
Vit_B12_pg/Ml	
*MTHFR*_C677T_wild_type	*MTHFR*_C677T_wild_type
*MTHFR*_C677T_heterozygous	
*MTHFR*_C677T_mutant	*MTHFR*_C677T_mutant
*MTHFR*_1298_wild_type	
*MTHFR*_1298_heterozygous	
*MTHFR*_1298_mutant	*MTHFR*_1298_mutant
*TYMS*_28bp_wild_type	
*TYMS*_28bp_heterozygous	*TYMS*_28bp_heterozygous
*TYMS*_28bp_mutant	
*TYMS*_6bp_wild_type	*TYMS*_6bp_wild_type
*TYMS*_6bp_heterozygous	*TYMS*_6bp_heterozygous
*TYMS*_6bp_mutant	
*MTRR*_A66G_wild_type	
*MTRR*_A66G_heterozygous	*MTRR*_A66G_heterozygous
*MTRR*_A66G_mutant	
*MTR*_A2756G_wild_type	
*MTR*_A2756G_heterozygous	
*MTR*_A2756G_mutant	*MTR*_A2756G_mutant
*RFC1*_A80G_wild_type	
*RFC1*_A80G_heterozygous	*RFC1*_A80G_heterozygous
*RFC1*_A80G_mutant	
*DNMT3B*-149C>T_wild_type	
*DNMT3B*-149C>T_heterozygous	*DNMT3B*-149C>T_heterozygous
*DNMT3B*-149C>T_mutant	*DNMT3B*-149C>T_mutant
*DNMT3B*-579G>T_wild_type	*DNMT3B*-579G>T_wild_type
*DNMT3B*-579G>T_heterozygous	*DNMT3B*-579G>T_heterozygous
*DNMT3B*-579G>T_mutant	*DNMT3B*-579G>T_mutant

### Semantic connectivity map

An existing mapping method [28,29] was used to highlight through a graph the most important links among variables, using a mathematical approach based on an artificial adaptive system called Auto Contractive Map-Auto-CM algorithm. The Auto Contractive Map (Auto-CM) is a special kind of Artificial Neural Network able to find, by a specific data mining learning algorithm, the consistent patterns and/or systematic relationships and hidden trends and associations among variables. After the training phase the weights developed by Auto-CM are proportional to the strength of associations of all variables each-other. The weights are then transformed in physical distances. Variables couples whose connection weights are higher become nearer and vice versa. A simple mathematical filter represented by minimum spanning tree is applied to the distances matrix and a graph is generated. This allows seeing connection schemes among variables and detecting variables acting as “hubs”, being highly connected. This matrix of connections preserves non linear associations among variables and captures connection schemes among clusters. After the training phase, the weights matrix of the Auto-CM represents the warped landscape of the dataset. Subsequently, a simple filter to the weights matrix of the Auto-CM system was applied to obtain a map of the main connections between the variables of the dataset and the basic semantic of their similarities, defined connectivity map as detailed elsewhere [28,29]. The dataset data were coded as shown if Figure 2 for genotypes. We transformed the three biochemical variables (folates, hcy, and vitamin B12) in six input variables constructing for each of the variable, scaled from zero to 1, its complement, as detailed elsewhere [60]. AutoCM ANN was designed by M Buscema at the Semeion Research Center in Rome, and developed in specific research softwares (AutoCM - Auto Contractive Map, Semeion software #46, version 6.0; Modular Auto-Associative ANN, Semeion software #51, version 18.1).

## Results

### Classification performances with ANNs

Tables 4 and 5 show the results in the two selected strategies of validation (K-Fold and Training and Testing with random Split, respectively) and using all the 30 variables in the dataset as input vectors. Generally speaking the classification capabilities of all the algorithms are poor (from 50% to 65% in general accuracy) and sometimes similar, except the Sine Net (71% of general accuracy). The conclusion could be: there is no evidence of correlation between these variables and AD, at least in this dataset. However, the application of TWIST algorithm to eliminate noisy variables before the main test of pattern recognition allowed the selection of 16 attributes (listed in Table 3). First, we have applied the K-Fold protocol to the global dataset to verify if the 16 attributes selected by TWIST may improve the performances of the learning machines already applied to the original dataset. Table 6 shows the results. The most of learning machines improve dramatically their performances (up to 70% and more of global accuracy) and both the Semeion ANNs reach up the 77% of global accuracy. Consequently, two of the tested algorithms were able to find a good correlation between some variables and AD, once noisy attributes were removed.

**Table 4:** Results of K-Fold protocol using all the 30 variables.

**30x2 K-Fold=10**	**Control**	**Alzheimer**	**A. Mean**	**W. Mean**	**Error**
**FF_Bp^a^**	**65.00%**	**65.00%**	**65.00%**	**65.00%**	**28**
**FF_Sn^a^**	**60.00%**	**65.00%**	**62.50%**	**62.50%**	**30**
Logistic	65.00%	57.50%	61.25%	61.25%	31
RotationForest	60.00%	60.00%	60.00%	60.00%	32
SMO	60.00%	57.50%	58.75%	58.75%	33
J48	62.50%	52.50%	57.50%	57.50%	34
MLP	60.00%	55.00%	57.50%	57.50%	34
NaiveBayes	77.50%	35.00%	56.25%	56.25%	35
RandomForest	62.50%	50.00%	56.25%	56.25%	35
IBk	60.00%	50.00%	55.00%	55.00%	36
AdaBoostM1	65.00%	40.00%	52.50%	52.50%	38
KStar	60.00%	45.00%	52.50%	52.50%	38
Bagging	55.00%	45.00%	50.00%	50.00%	40
LogitBoost	50.00%	47.50%	48.75%	48.75%	41
BayesNet	87.50%	7.50%	47.50%	47.50%	42

a Semeion ANNs are in bold

**Table 5:** Results of random split protocol using all the 30 variables.

**30x2 Tr-Ts**	**Control**	**Alzheimer**	**A. Mean**	**W. Mean**	**Error**
**FF_Sn^a^**	**72.50%**	**70.00%**	**71.25%**	**71.25%**	**23**
Logistic	70.00%	57.50%	63.75%	63.75%	29
LogitBoost	57.50%	70.00%	63.75%	63.75%	29
**FF_Bp^a^**	**72.50%**	**52.50%**	**62.50%**	**62.50%**	**30**
NaivBayes	67.50%	45.00%	56.25%	56.25%	35
AdaBoost	52.50%	55.00%	53.75%	53.75%	37
MLP	65.00%	37.50%	51.25%	51.25%	39
RandomForest	72.50%	30.00%	51.25%	51.25%	39
BayesNet	100.00%	0.00%	50.00%	50.00%	40
J48	62.50%	37.50%	50.00%	50.00%	40
SMO	62.50%	37.50%	50.00%	50.00%	40
IBk	65.00%	32.50%	48.75%	48.75%	41
KStar	57.50%	37.50%	47.50%	47.50%	42
Bagging	45.00%	45.00%	45.00%	45.00%	44
RotationForest	60.00%	30.00%	45.00%	45.00%	44

a Semeion ANNs are in bold

**Table 6:** Results with the K-Fold protocol using the 16 variables selected by TWIST algorithm.

**Twist 16x2 K-Fold=10**	**Control**	**Alzheimer**	**A. Mean**	**W. Mean**	**Error**
**FF_Bp^a^**	**80.00%**	**75.00%**	**77.50%**	**77.50%**	**18**
**FF_Sn^a^**	**82.50%**	**72.50%**	**77.50%**	**77.50%**	**18**
IBk	77.50%	65.00%	71.25%	71.25%	23
MLP	67.50%	72.50%	70.00%	70.00%	24
RotationForest	70.00%	70.00%	70.00%	70.00%	24
J48	57.50%	70.00%	63.75%	63.75%	29
Logistic	62.50%	60.00%	61.25%	61.25%	31
SMO	57.50%	65.00%	61.25%	61.25%	31
KStar	67.50%	52.50%	60.00%	60.00%	32
LogitBoost	62.50%	52.50%	57.50%	57.50%	34
NaiveBayes	70.00%	45.00%	57.50%	57.50%	34
RandomForest	70.00%	45.00%	57.50%	57.50%	34
AdaBoostM1	57.50%	52.50%	55.00%	55.00%	36
Bagging	60.00%	42.50%	51.25%	51.25%	39
BayesNet	87.50%	7.50%	47.50%	47.50%	42

a Semeion ANNs are in bold

But K-Fold protocol is not a trustable strategy as shown in [51]. TWIST, in fact, generates also two new subsets (with the selected variables) with a similar density of probability distribution [51]. That means that the two subsets are statistically equivalent and each of them is also equivalent to the global dataset. The K-Fold protocol has not this capability, and for this reason its results are an average whose variance could be very high. For this reason as a second step we have applied the same learning machines to the two subsets generated directly by TWIST. The results are shown in Table 7. In this case the performances of all the learning machines are still improved and some of them (Sine Net, IBk and Back Prop) show to be able to be used as optimal predictors of AD (Table 7).

**Table 7:** Results with the two subsets generated by TWIST using the 16 variables.

**Twsit 16x2 (Tr-Ts)**	**Control**	**Alzheimer**	**A. Mean**	**W. Mean**	**Error^b^**
**FF_Sn^a^**	**92.67%**	**94.99%**	**93.83%**	**93.80%**	**5**
IBk	96.00%	89.72%	92.86%	92.33%	6
**FF_Bp^a^**	**86.67%**	**82.58%**	**84.62%**	**84.34%**	**13**
MLP	78.00%	75.19%	76.59%	76.66%	19
J48	74.67%	74.94%	74.80%	74.81%	20
RotationForest	75.33%	72.06%	73.69%	72.63%	22
Logisitc	70.67%	69.92%	70.30%	70.08%	24
KStar	68.00%	69.67%	68.84%	67.90%	26
RandomForest	86.67%	42.23%	64.45%	63.49%	29
AdaBoost	72.67%	46.74%	59.70%	58.70%	32
Bagging	66.67%	56.27%	61.47%	58.76%	33
LogitBoost	68.67%	49.62%	59.15%	58.38%	33
NaiveBayes	71.33%	49.62%	60.48%	59.14%	33
SMO	56.00%	63.03%	59.52%	59.91%	33
BayesNet	50.00%	50.00%	50.00%	44.88%	44

a Semeion ANNs are in bold,

b The number of errors is the summation of the error performed in testing phase using both the subsets.

### Semantic connectivity map

**Figure 3:**
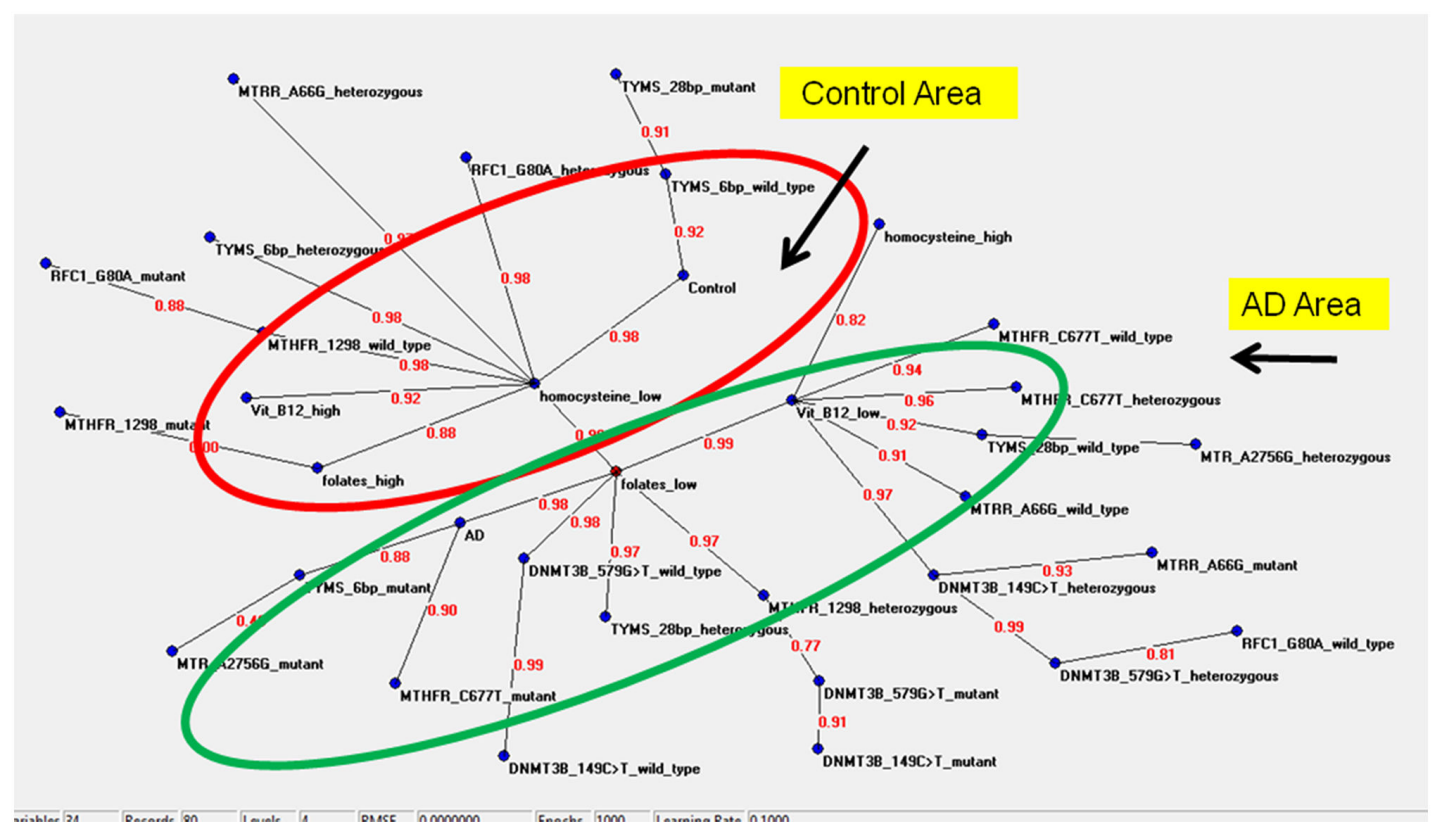
Semantic connectivity map obtained with Auto-Cm System. The figures on the arches of the graph refer to the strength of the association between two adjacent nodes. The range of this value is from 0 to 1.

Figure 3 shows the semantic connectivity map obtained with the application of the Auto-CM system. Variables which have the maximal amount of connections with other variables are called “hubs” of the system. In order to better understand the meaning of the connections a numerical value is applied to each edge of the graph. This value, deriving from the original weight developed by Auto-CM during the training phase scaled from 0 to 1, is proportional to the strength of the connections among two variables. Results clearly indicated that AD cases can be visually separated from the controls, and particularly it was possible to visualize an AD area characterized by low folates, low vitamin B12, high hcy and several risk genotypes, and a control area characterized by low hcy, high folates, high vitamin B12, and several protective genotypes (Figure 3). Moreover, by means of Auto-CM, it is possible to obtain not only the direction of the association as provided by standard statistical analyses, but importantly also the strength of this association (link strength = ls). For example, reduced folates were strongly (ls=0.98) related to AD as it was the *MTHFR* 677 mutant (TT) genotype (ls=0.90) and the *TYMS* 1494 6bp mutant (-/-) genotype (ls=0.88). Reduced folates were also closely linked to low levels of vitamin B12 (ls=0.99), and this condition was linked to increased hcy levels (ls=0.82). Several genotypes were also connected to low vitamin B12 levels (Figure 3). Concerning control subjects they resulted strongly connected with the *TYMS* 1494 6bp wild-type (+/+) genotype (ls=0.92) and with reduced hcy levels (ls=0.98) which in turn were connected with high vitamin B12 and folate levels, as well as with several genotypes (Figure 3).

## Discussion

Both prospective and retrospective studies have suggested a possible link among folate, hcy, and vitamin B12 levels and AD risk [3–13]. Moreover, there is indication from genetic association studies, GWAS, and meta-analyses of the literature, suggesting that polymorphisms of genes involved in one-carbon metabolism might represent AD genetic susceptibility factors [14–17]. In the present study we selected 40 late-onset AD subjects and 40 age and sex matched controls to see if ANNs were able to discriminate between those two conditions using a set of data that included the circulating values of folate, hcy and vitamin B12 and 27 different genotypes generated by nine biallelic polymorphisms of genes involved in one-carbon metabolism.

Through TWIST system, we established a consistent possibility to predict the status of being an AD or a control subject on the basis of 16 selected variables (Table 3) that allowed to reach up to 90% global accuracy to some of the used learning machines (Table 7), this meaning that the selected variables contained specific information to discriminate between the two conditions. In particular, folate and hcy values, as well as *MTHFR* 677CC, *MTHFR* 677TT, *MTHFR* 1298CC, *TYMS* 28bp 2R/3R, *TYMS* 1494 6bp +/+, *TYMS* 1494 6bp +/-, *MTRR* 66AG, *MTR* 2756GG, *RFC1* 80GA, *DNMT3B* -149CT, *DNMT3B* -149TT, *DNMT3B* -579GG, *DNMT3B*-579GT, and *DNMT3B* -579TT genotypes resulted the most important variables for discriminating between AD and control subjects (Table 3). Most of these variables, such as folate, hcy, *MTHFR, MTRR*, and *RFC1* genotypes, had been previously associated with AD risk (reviewed in 2), but others, including *TYMS* and *DNMT3B* genotypes, were not associated with disease risk when considered independently from the others [8,27]. The present study represents the first attempt to use ANNs to understand the complex relationship between one-carbon metabolism and AD, and at best of our knowledge also the first attempt to evaluate the combined effect of 30 different variables in this pathway to AD pathogenesis. ANNs provided a valuable tool to evaluate the whole pathway and to unravel the links among studied variables as shown in the semantic connectivity map (Figure 3). Particularly, the semantic connectivity map obtained by means of the Auto-CM system revealed already known connections as well as novel ones (Figure 3). It is not surprising that reduced folates resulted the most related variable linked to AD (ls=0.98), since several literature papers observed reduced blood folate levels in AD patients with respect to controls [2,6,8,11,13]. Moreover, the *MTHFR* 677TT genotype was closely linked to AD (ls=0.90), and this is also known from the literature [8,15], as it is known that the effect of this mutant genotype is exacerbated under conditions of reduced folates that impair protein stability and activity [2]. The observed strong link between reduced folates and reduced vitamin B12 levels (ls=0.99) is also known [1,8], and this condition is likely to foster an increase in hcy levels (ls=0.82) that is often seen in AD individuals [1–10], likely because of vitamin B12 is a cofactor required by the MTR/MTRR complex during the conversion of hcy to methionine (Figure 1). Indeed, several genotypes such as those generated by *MTHFR, MTR*, and *MTRR* polymorphisms are likely to contribute to vitamin B12 levels, but also those in *TYMS* and *DNMT3B* genes for the existence of feedback inhibitory loops in the pathway [2]. Very interesting and unexpected is the link between the *TYMS* 1494 6bp -/- genotype and AD (ls=0.88), that was paralleled by a strong link between the the *TYMS* 1494 6bp +/+ genotype and the condition of being a healthy control (ls=0.92) (Figure 3). At best of our knowledge the present is the first report of a possible contribution of this polymorphism to AD risk. The *TYMS* 1494 6bp ins/del polymorphism impairs the TYMS mRNA stability and is often studied in conjunction with the 28bp repeat polymorphism in the promoter of the gene that affects gene expression levels [2]. Previous reports by us revealed a borderline significant difference (*P* =0.08) in the distribution of *TYMS* 28bp 2R and 3R alleles and related genotypes between late onset AD subjects and healthy matched controls [8]. Taken overall, present and previous data by us suggest that *TYMS* might be another candidate gene of the one-carbon metabolic pathway deserving further investigation in AD genetic association studies. Indeed, impairments of TYMS might shift the metabolic pathway toward DNA methylation (Figure 1), and favour epigenetic processes that are increasingly linked to AD pathogenesis [2].

Among factors tightly linked to controls there is low hcy (ls= 0.98), which is linked to high folates and high vitamin B12. This is not surprising since several authors previously observed reduced hcy and increased folate and vitamin B12 levels in controls with respect to AD subjects [1–13]. Several gene polymorphisms are linked to those conditions. For example, *MTHFR* 1298 homozygous genotypes are in the control area of the map. This is not surprising because of the effect of this polymorphism is often reported to be opposite to that of the *MTHFR* 677C>T one in AD risk, and has been often suggested to be a protective factor for AD [61,62].

None of the genotypes generated by *DNMT3B* polymorphisms have been directly linked to AD or control conditions (Figure 3), and this partially confirms the results of a previous genetic association study by us [27]. However, those genotypes seem to interact with others and play a role in determining folate and vitamin B12 levels (Figure 3), suggesting that their contribution to AD risk might be completely different when evaluated in combination with other variables of the pathway.

Several factors, and particularly medicaments and dietary supplements, may alter significantly the one-carbon metabolism. One example is that of metformin, an antidiabetic and gerosuppressant drug that has been suggested to work against AD, even if with conflicting results [63,64]. Indeed, metformin was shown to impair one-carbon metabolism in a manner similar to the antifolate class of chemotherapy drugs [65,66]. Other factors that could affect folate metabolism in aged individuals are dietary supplements containing folate, B-vitamins, or similar [67]. In order to minimize the effect of polymedication in our cohort of subjects, biochemical measurements of folate, hcy, and vitamin B12 were performed during the first visit and most of the subjects were not regularly taking supplements known to interfere with this pathway. In the case of individuals taking medicaments or supplements known or suspected to interfere with one-carbon metabolism, they were interrupted for one month before blood drawings. If this was not possible, the subject was not enrolled for the study.

Present results are indicative of a possible contribution of one-carbon metabolism variables as an additional tool to help during AD diagnosis. At this regard, a recent report from the Vienna Transdanube aging study suggests that high cortisol and low folate levels are the only routine blood tests predicting probable AD after age 75-years, thereby stressing on the utility of a deeper understanding of folate metabolism in AD pathogenesis [68]. Indeed, authors followed 493 persons who were cognitively healthy at baseline for a period of 90 months, and observed that a serum folate increment of 10 ng/mL reduced the risk of switching to probable AD to one-third [68]. Present data revealing that reduced folates are the most related variable linked to AD in our cohort are in strong agreement with that study, but we must stress that biochemical markers alone can be useful, but not sufficient to fully discriminate between AD and control subjects. However, their combination with neural correlates and imaging data, as well as with other markers of the disease such as cerebrospinal fluid markers, might be really useful within this context.

## Conclusions

The present study represents the first attempt to use ANNs the understand folate metabolism in AD and healthy matched control subjects, and reveals the importance to evaluate this pathway as a whole rather than to take into consideration its components one at once. Among 30 initial variables of the pathway, 16 of them seem to contain significant information to discriminate between AD and control subjects in our cohort, and the semantic connectivity map here generated reveals both already known and novel connections among variables and disease risk (Figure 3). Of particular interest are variables, such as *TYMS* and *DNMT3B* genotypes, that albeit not previously detected in genetic association studies might play a significant contribution when considering the complexity of interactions with other variables of this pathway. Though we achieved good results using ANNs for our small dataset, results are not necessarily generalizable to other populations but need to be validated independently in future studies. Differences might arise from a population to another one, due to different dietary habits or to a different distribution of the studied polymorphisms and other geographic factors. However, our system is able to understand the connections among studied variables and those of relevance in a particular dataset. The addiction of other variables, such as brain volume, DNA methylation content, DNA damage, and so on, coupled with the possibility to graphically visualize the strengths of connections among all the studied variables, could be a helpful and timely tool to unravel the link between folate metabolism and AD, particularly in a period when nutritional supplementation has been often suggested as a preventative strategy to delay epigenetic modifications linked to the onset of age-related disease such as AD [69].
